# "Drunk or Dying" Cases

**Published:** 1903-02-28

**Authors:** 


### EDITOR'S LETTER-BOX.

**[Our Correspondents are reminded that prolixity is a great bar to publication, and that brevity of style and conciseness of statement greatly facilitate early insertion.]**

### "DRUNK OR DYING" CASES.

The Hon. Sydney Holland, Chairman of the London Hospital, writes, under date of February 23rd, 1903: It is, of course, a pity to waste time in answering a statement which you say you never made. I wrote that you stated that "at the London Hospital no attempt had been made to provide beds for what are called 'drunk or dying' cases." Your actual words were, "no attempt whatever has been made to provide temporary beds for these doubtful cases." Do you rely for your argument on the words "temporary beds"? My reply was that in addition to an observation room we provide, it is true, not *temporary* beds, but *permanent* beds (which *can be used for temporary periods*) for such cases, and, if noisy, that we have a ward for them, but a ward not reserved specially and only for these cases. I

have no desire to "beg the question" either this or any other, as you write, and no one can in fairness say that my letter did so. I will now answer your catechism.

1. Does Mr. Holland deny that our representative ascertained on inquiry that no beds are specially provided for drunk or dying cases?

I know nothing of what your representative ascertained. In my letter I replied categorically to this, and said that no beds were reserved specially for these cases—the whole hospital is open to them. This, at any rate, was not "begging the question."

2. Does Mr. Holland maintain that there is no harm or risk to a certain class of cases by not having such special beds?

Certainly I do, and I said so definitely in as plain English as I could command.

3. If such beds were provided, does he maintain that the receiving-room officers would not admit certain doubtful cases which may at present be sent away?

Yes, I do; the provision of such beds ought not to make any difference at all. The receiving-room officer ought not to admit a drunk case, and ought not to send away either a dying or a doubtful case, and a provision of a temporary ward ought not to make any difference whatever in his action. This is what I stated plainly.

4. Does he maintain that the St. Mary's case and the Middlesex case referred to in our article were not doubtful cases?

I never said anything of the sort—I did not deal with the St. Mary's case, which was not referred to in the article under reply, entitled a "Grave Scandal"—but I will. In the St. Mary's case, as reported, the receiving-room officer made a mistake; he failed to diagnose a fractured skull, and a similar mistake was made in the Middlesex case. At both hospitals, if the diagnosis had been correct, the cases would have been admitted. In the article entitled "A Grave Scandal," you referred to a St. Thomas's case as proving your point. This case is now dropped out of your catechism, and I do not wonder, as in that case the statement of the coroner was, that if the patient had been properly examined, she would have been admitted. This bears out exactly what I have said of all these cases.

5. Does Mr. Holland contend that it is justifiable to subject patients in a surgical ward to the disturbance and unpleasantness caused by admitting at any time a drunk or dying case to a vacant bed?

Again, I answered this distinctly, and it is, I should have thought, unnecessary to make me answer again. My reply is that it is not right to assume that all these emergency cases are either noisy or unpleasant to other patients. If they are noisy we send them to a ward reserved for noisy cases, but as I have said before, not specially reserved for these cases. Almost all anæsthetic cases when recovering are noisy, and their language is often what may be described as "nervous English," but other patients have to put up with this as one of the penalties, I fear, of hospital treatment. But the curious part of your criticism of my letter and of the catechism to which you now subject me is that you omit any reference to the fact which I set out in my letter that *finding that these cases had often caused inconvenience and annoyance to other patients we are building* 12 *rooms* for 24-hour cases, and that I told this to the Editor of THE HOSPITAL myself before the article appeared. How can I answer more plainly, or how have I begged the question?

I do not wish to shirk for myself or for my committee any responsibility whatever. We do what we think right and accept the responsibility for our action. You blame me for not defending a receiving-room officer. I should be doing very wrong if I defended a man when he had made a mistake, a mistake very painful to himself, and I should be doing equally wrong if I blamed him too severely. Every case has to be judged on its merits. I repeat that we have taken every precaution against an occurrence of such mistakes as you allude to. We elect six of the best men we have to hold the posts of receiving-room officers. We are certain that they will always do their very best, and we are equally certain that it is inevitable that from time to time they will, in common with the leaders in their profession, make mistakes. It is only 12 years ago that the most eminent man in the profession diagnosed me as a man giving way to the temptation of drink when I went to consult him about the result of a concussion to my poor brain—this in his own quiet consulting-room and not in the hurry of a receiving-room. It is in my opinion—for what it is worth—wrong to argue that if these wards were provided our men would be less likely to make mistakes than now, when the whole hospital is open for the reception of these cases, and I think it is an abuse of fair comment to call the non-provision of these wards a "Grave Scandal."

*** THE POSITION OF THE LONDON HOSPITAL.

Mr. Sydney Holland proves either too much or too little. He contradicts himself, and shows the danger of a layman expressing strong opinions upon a question which depends mainly on the possibility of an accurate diagnosis in certain circumstances. The whole medical profession will agree that Mr. Sydney Holland is not justified and, indeed, is even wrong in attempting to make the receiving-room officers responsible for the consequences which result from the non-admission of a "drunk or dying" case in the absence of a provision of emergency beds at the hospital where admission has been refused. Mr. Holland ought to know, and if he does know he ought to withdraw much that he states in the above letter, that unless many of the "drunk or dying" cases are kept under observation for a number of hours no medical man can make a certain diagnosis. His own case, as he states it, proves this to be so. Mr. Holland admits that the London Hospital contains no emergency beds; that the practice is to admit to one of the general wards every "drunk or dying" case brought to the hospital; and that "Richmond Ward is reserved for noisy cases, but not specially for these noisy ones." We have ascertained by inquiries at the hospital that "drunk or dying" cases at the London do not average more than one each night, from which it would appear that the patients in any surgical ward at the London Hospital are liable to be upset nightly by the admission of one of these cases, with the consequent disagreeables and dangers to the cleanliness and hygiene of the ward which they involve. Some of them may also be liable to have noisy cases admitted because Richmond Ward can only, we presume, be devoted to one sex. "Drunk or dying" cases often arrive late at night or in the early morning. They are usually brought in by the police probably they smell strongly of alcohol, are unconscious or noisy, or both, exhibit symptoms of vomiting, are sometimes horribly dirty, and even alive with vermin. Yet this is the position Mr. Sydney Holland endeavours to justify in his letter. Such an attitude does not show any regard to what is due to the other inmates of the hospital.

Again, what point is there in pleading that a new building is being erected with twelve rooms for these cases when the necessity for an immediate temporary accommodation is shown to be urgent, and can be readily made without appreciable cost or difficulty? Where emergency beds have been provided they are usually of the simplest character—two rooms, one for men and one for women, in the basement or some part of the building, where a noisy patient will not prove a nuisance. The accommodation is rough but sufficient, and all the surroundings are such that, however dirty the patient or disagreeable the case, no injury can result to anybody, and the whole place can be purified with the utmost facility. It should be, and no doubt is, possible, for the authorities of the London Hospital, if they have the will, to provide such accommodation for emergency cases at any time within twenty-four hours. To continue the present system an hour longer than necessary is to emphasise the fact, that the London Hospital, or any other hospital which continues such a system, must lack adequate administrative control, and needs, as most authorities who have considered the matter have known for years, a medical superintendent to be in constant charge of the whole internal administration, including the supervision of the admission of patients. Such are the facts, and it is for the authorities of the London Hospital to determine what position they will occupy as hospital administrators and custodians of the interests of the public. The decision which they now give in regard to the immediate provision or non-provision of emergency beds for "drunk or dying" cases of both sexes will decide this.

We regret very much that Mr. Holland should so strenuously resist this necessary reform, which has been called for by the medical profession and public opinion for so many years. We regret, too, that he should have interpreted the article in The Hospital as aimed at the London Hospital, for that is exactly what we endeavoured to avoid, for in that article we did not deal with one hospital, but with all the great hospitals with medical schools, that is, eight out of twelve, which have failed to provide emergency beds. It is the callousness of some of the lay managers of the hospitals in regard to this question which we have attacked, for the sake of the public, the honour of the hospitals, the resident medical staff, and the whole of the medical profession. We are confident that this callousness of the lay managers is due to their failure to appreciate the actual position, owing to their want of medical knowledge. This leads us to hope, that before many weeks are over, every one of the eight hospitals in question will have provided half a dozen emergency beds, say three for men and three for women, which cannot now be left unprovided, by any great hospital, without the loss of reputation and public sympathy.—[Ed. The Hospital.]

